# An Interpretable Multi-Objective Machine Learning Framework for In Silico Prioritization of Anti-*Staphylococcus aureus* Antimicrobial Peptides

**DOI:** 10.3390/diagnostics16152430

**Published:** 2026-08-01

**Authors:** Jianguo Xu, Donghua Yang, Qingyong Zheng, Tengfei Li, Yating Cui, Jinhui Tian

**Affiliations:** 1 Evidence-Based Medicine Center, School of Basic Medical Sciences, Lanzhou University, Lanzhou 730000, China; xujg15@126.com (J.X.); 120220905900@lzu.edu.cn (D.Y.); easonzz@foxmail.com (Q.Z.); cuiyating112@163.com (Y.C.); 2 Population Health Research Institute, Hamilton, ON L8L 2X2, Canada; 3 Qinghai University Affiliated Hospital, Xining 810000, China; 4 School of Nursing, Chinese Academy of Medical Sciences and Peking Union Medical College, Beijing 100730, China; ltf980102@163.com; 5 Key Laboratory of Evidence-Based Medicine of Gansu Province, Lanzhou University, No. 199, Donggang West Road, Lanzhou 730000, China

**Keywords:** antimicrobial peptides, *Staphylococcus aureus*, MRSA, machine learning, interpretability, virtual screening, hemolysis prediction

## Abstract

**Background**: *Staphylococcus aureus*, including methicillin-resistant lineages, is a leading cause of device- and catheter-related infection, and rising resistance motivates the search for antimicrobial peptides (AMPs) with strong anti-staphylococcal activity and low host toxicity. Machine learning can prioritize candidate peptides. However, the literature-derived AMP datasets are prone to homology-driven optimism, and computational studies frequently overstate their translational reach. **Methods**: We curated 4007 deduplicated *S. aureus*-active AMP records and 582 binary-labeled hemolysis records. Each peptide was encoded with a transparent 538-dimensional physicochemical and compositional feature vector. Five classifiers and five regressors were evaluated for four endpoints (potency classification, log_10_ MIC regression, hemolysis classification, normalized hemolytic index) under both conventional random 5-fold cross-validation and homology-aware cross-validation, in which sequences were clustered by 3-mer similarity and whole clusters were confined to single folds. Class imbalance was handled by class weighting. Model behavior was interpreted with SHAP and Fisher-exact k-mer enrichment, and candidates were ranked by a multi-objective score that combines the independently trained heads. **Results**: Under homology-aware validation, performance was lower than under random splitting, as expected. Potency classification reached an AUROC of about 0.71 (Random Forest), compared with 0.797 under random cross-validation. Hemolysis classification remained strong at AUROC 0.90 (95% CI 0.88 to 0.93), which indicates that its high accuracy is not a homology leakage artifact. MIC regression was modest (homology-aware R^2^ 0.17, Spearman ρ 0.39) and is therefore treated only as a rank-ordering signal. SHAP and k-mer analyses recovered interpretable structure–activity relationships. Net positive charge and amphipathicity drove potency, whereas bulk hydrophobicity drove hemolysis. Applying the pipeline to a generated pool prioritized 20 candidates. Nearest-neighbor analysis shows that these are close optimized variants of known potent scaffolds, with a median identity of 95% to a known peptide, rather than novel sequences. **Conclusions**: We present an interpretable, honestly benchmarked multi-objective pipeline that optimizes known anti-*S. aureus* AMP scaffolds toward lower predicted hemolysis. The prioritized peptides are computational hypotheses for future synthesis and experimental testing. Their low predicted hemolysis reflects a selection criterion rather than validated safety, and cross-species selectivity was not assessed.

## 1. Introduction

*Staphylococcus aureus*, including methicillin-resistant lineages (MRSA), is the dominant Gram-positive pathogen in catheter- and device-related infections. Biofilm formation on polymeric surfaces shields the bacteria from host defenses and from antibiotics [1,2,3,4]. Early laboratory recognition of bloodstream infection remains important for outcomes, and simple screening scores have been proposed to speed it [5]. Rapid molecular and biosensor assays for MRSA continue to be developed in parallel [6]. With antimicrobial resistance projected to cause a rising toll worldwide [7,8], and with the challenge extending to carbapenem-resistant Gram-negative organisms as well [9], new antimicrobial agents are actively sought [10]. Antimicrobial peptides (AMPs) have attracted interest as membrane-active agents with a comparatively low propensity to select for resistance [11,12].

The translation of AMPs is nonetheless constrained by real liabilities that computational work should not gloss over. Many AMPs are hemolytic or cytotoxic. Many are susceptible to proteolytic degradation, bind serum proteins, and are costly to synthesize, and only a minority have reached clinical use [11,12]. A useful computational contribution therefore lies less in declaring new hits than in transparently prioritizing and optimizing peptides while making the limitations explicit.

Machine learning has been applied widely to AMP prediction. Methods range from random forests [13,14] to convolutional and recurrent networks [15,16], protein language models [17,18], and generative design platforms [19,20,21,22]. Related matrix-factorization approaches have also been used for computational drug repurposing [23]. Three gaps remain relevant here. First, most predictors are taxon-agnostic, labeling sequences as AMP or non-AMP, whereas anti-staphylococcal activity is the clinically relevant endpoint for device infection. Second, activity and host toxicity are rarely optimized together within a single interpretable framework [24]. Third, and most important for reliability, the literature-derived AMP datasets contain families of near-identical sequences. Without homology-aware evaluation, reported performance can be optimistic, and candidate novelty judged by exact-match identity can be illusory.

To address these critical gaps, we developed a targeted, in silico multi-objective screening framework. By curating an *S. aureus*-specific dataset and utilizing a transparent 538-dimensional feature space, interpretable tree-based models were trained to predict both antimicrobial potency and hemolytic toxicity. Crucially, to ensure robust generalization and address the risk of dataset redundancy, all models were benchmarked using both random and homology-aware cross-validation. Furthermore, we integrated SHAP analysis and k-mer enrichment to map structure–activity relationships, ultimately employing a multi-objective scoring function to prioritize and optimize candidate sequences. By tracing the nearest-neighbor identities of these top-ranked variants to known potent scaffolds, this work provides an interpretable prioritization tool and a set of experimentally testable hypotheses, not as a validated discovery.

## 2. Materials and Methods

### 2.1. Data Sources and Curation

**Figure 1:**
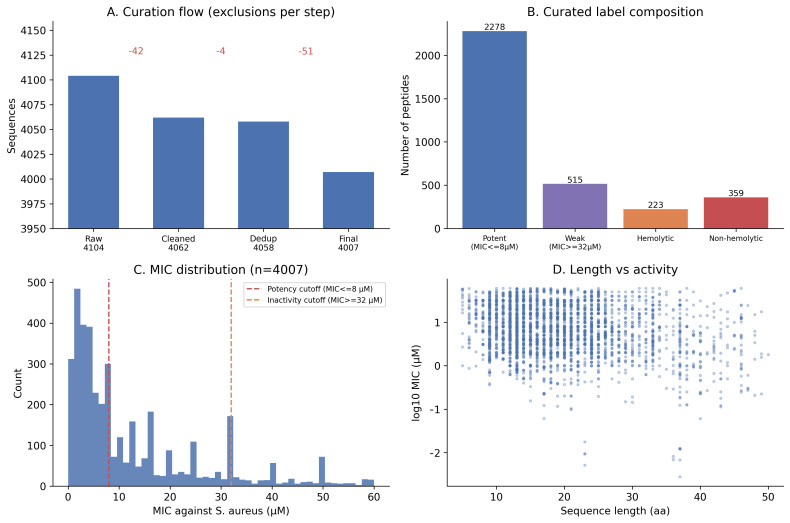
Dataset construction and characteristics. (**A**) Sequences retained and excluded at each curation step. (**B**) Curated label composition (potent, weak, hemolytic, and non-hemolytic counts). (**C**) MIC distribution with classification cutoffs. (**D**) Length versus log10 MIC.

Anti-staphylococcal AMPs were extracted from dbAMP [25]. Records were restricted to standard amino acids and lengths of 5 to 50 residues. When a sequence appeared multiple times with different reported MICs, which arises from different strains, media, or laboratories, we retained the lowest MIC to reflect intrinsic optimal activity. This deduplication and its effect on record counts are reported in Figure 1A. Strain annotations, including MRSA and MSSA status where available, were retained as metadata but were not used as modeling labels, because the labeled subsets are too small and unevenly distributed for reliable strain-specific training. This is stated as a limitation. The final modeling set comprised 4007 distinct *S. aureus* sequences.

For classification, sequences were labeled potent (MIC at most 8 µM, *n* = 2278) or weak (MIC at least 32 µM, *n* = 515), leaving an intermediate band unlabeled to sharpen the decision boundary. We use these thresholds as pragmatic operating points rather than as formal CLSI breakpoints [26], because peptide MIC distributions do not map onto conventional small-molecule breakpoint logic. The sensitivity of conclusions to this choice is limited, because MIC is used mainly for ranking. Hemolysis data were compiled from an integrated set of 1273 peptide-concentration records. Because assays differ in red-blood-cell source, incubation, concentration, and format, we binarized to hemolytic (at least 30% lysis at up to 50 µg mL^−1^, *n* = 223) and non-hemolytic (at most 10% lysis at 50 µg mL^−1^ or above, *n* = 359), which yielded 582 unambiguous labels, and we treated the heterogeneity as an explicit source of noise. A normalized hemolytic index, NHI = H/(ln C + e), with H the percent lysis and C the concentration in µg mL^−1^, was computed as a coarse, monotone summary that down-weights high-concentration lysis. We make no claim that it fully harmonizes across assays, and we report it only as a secondary, exploratory endpoint.

### 2.2. Feature Engineering

We featurized each peptide using a comprehensive 538-dimensional array to capture multiple structural scales and biophysical properties crucial for membrane interaction. This included 20 features for amino-acid composition (AAC), 400 for dipeptide composition (DPC) [27], and 13 global physicochemical descriptors (e.g., sequence length, molecular weight, net charge at pH 7, mean Kyte–Doolittle hydrophobicity [28], Boman index [29], and maximum Eisenberg hydrophobic moment [30]).

Additionally, we extracted 105 Composition–Transition–Distribution (CTD) features based on the Dubchak scheme [31]. These features are highly relevant for modeling amphipathic structures, as they capture the frequency and distribution of transitions between polar and non-polar domains along the peptide backbone, effectively representing solvent accessibility and secondary structure propensities without requiring computationally expensive 3D folding simulations. We deliberately excluded complex protein-language-model embeddings from this primary model to ensure that every input dimension remained transparent and accessible for direct mechanistic interpretation.

### 2.3. Models, Imbalance Handling, and Cross-Validation

For each endpoint we evaluated logistic regression, linear SVM, Random Forest [32], XGBoost [33], and LightGBM [34] for classification, and ridge, linear SVR, Random Forest, XGBoost, and LightGBM for regression. Features were standardized within each training fold. Class imbalance was handled by class weighting. We additionally verified that synthetic minority oversampling (SMOTE) [35] gave statistically indistinguishable performance (Supplementary Table S4), and we adopt class weighting so that no synthetic, biologically implausible feature vectors enter training.

Two cross-validation schemes were used. The first is conventional random 5-fold cross-validation, stratified for classification. The second, which we treat as the primary estimate of generalization, is homology-aware cross-validation. Peptides were clustered by an alignment-free 3-mer overlap-coefficient greedy procedure at a threshold of 0.6, and entire clusters were assigned to single folds using StratifiedGroupKFold for classification or GroupKFold for regression. This ensures that near-identical or scaffold-related sequences cannot straddle the train–test boundary. Clustering reduced 2793 potency sequences to 1264 clusters, 582 hemolysis sequences to 297, and 4007 MIC sequences to 1683, which confirms substantial homology structure. For the two production classifiers, we additionally report out-of-fold confusion matrices, sensitivity, specificity, bootstrap 95% confidence intervals on AUROC, and reliability curves.

### 2.4. Interpretability Analyses

SHAP values were computed with TreeExplainer [36] on the production classifiers, analyzing mean absolute and signed contributions. We also computed amino-acid composition shifts between potent and weak pools, and Fisher-exact k-mer enrichment for k = 2 and 3 with Bonferroni correction. The global geometry of the feature space was visualized with UMAP [37] using a cosine metric. We treat UMAP strictly as a visualization and draw no inference about novelty or mechanism from it.

### 2.5. Multi-Objective Prioritization

The best model per endpoint (Random Forest for potency, MIC, and NHI; LightGBM for hemolysis) was retrained on all data and used to score candidates. The four heads are trained independently and combined only at scoring. We therefore describe the procedure as multi-objective prioritization rather than multi-task learning. Candidates were ranked by

D(x) = p(potent|x) · (1 − p(hemo|x)) · exp(−max(0, MIC_pred(x))/4).

A candidate pool was generated by point-mutating the most potent natural scaffolds and by random length-matched sampling. After standardization and filtering (potency probability at least 0.8, hemolysis probability at most 0.2, estimated MIC at most 4 µM, length 10 to 25, and no exact match to any known sequence), surviving peptides were clustered by 3-mer cosine distance, and the top-scoring representatives were retained. Because candidates are filtered on predicted hemolysis, their low predicted hemolysis is a property of the selection and not independent evidence of safety. For every retained candidate we computed the nearest-neighbor identity to the entire curated known-AMP set by global BLOSUM62 alignment, and we assessed how sensitive the ranking is to the exact form of D by perturbing its components. Analyses used Python (version 3.11.8) with fixed seeds (SEED = 42) and scikit-learn (version 1.4.1.post1), XGBoost (version 2.0.3), LightGBM (version 4.3.0), SHAP (version 0.45.0), imbalanced-learn (version 0.12.0), BioPython (version 1.83), and UMAP-learn (version 0.5.5).

## 3. Results

### 3.1. Dataset

The curated *S. aureus* set comprised 4007 peptides, with a median length of 17 residues and a median MIC of 7.07 µM. The hemolysis set yielded 582 binary labels (Figure 1). Clustering revealed extensive sequence homology, which motivates the homology-aware evaluation below.

### 3.2. Cross-Validation Benchmarking

**Figure 2:**
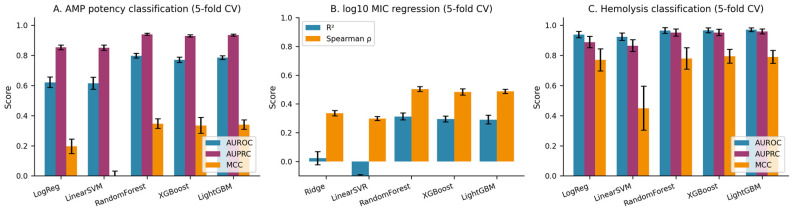
Five-fold cross-validation performance of the five models on (**A**) potency classification, (**B**) log_10_ MIC regression, and (**C**) hemolysis classification under conventional random splitting. Metrics include AUROC, AUPRC, and MCC for classification and R^2^ and Spearman ρ for regression. Error bars show plus or minus one standard deviation across folds.

**Figure 3:**
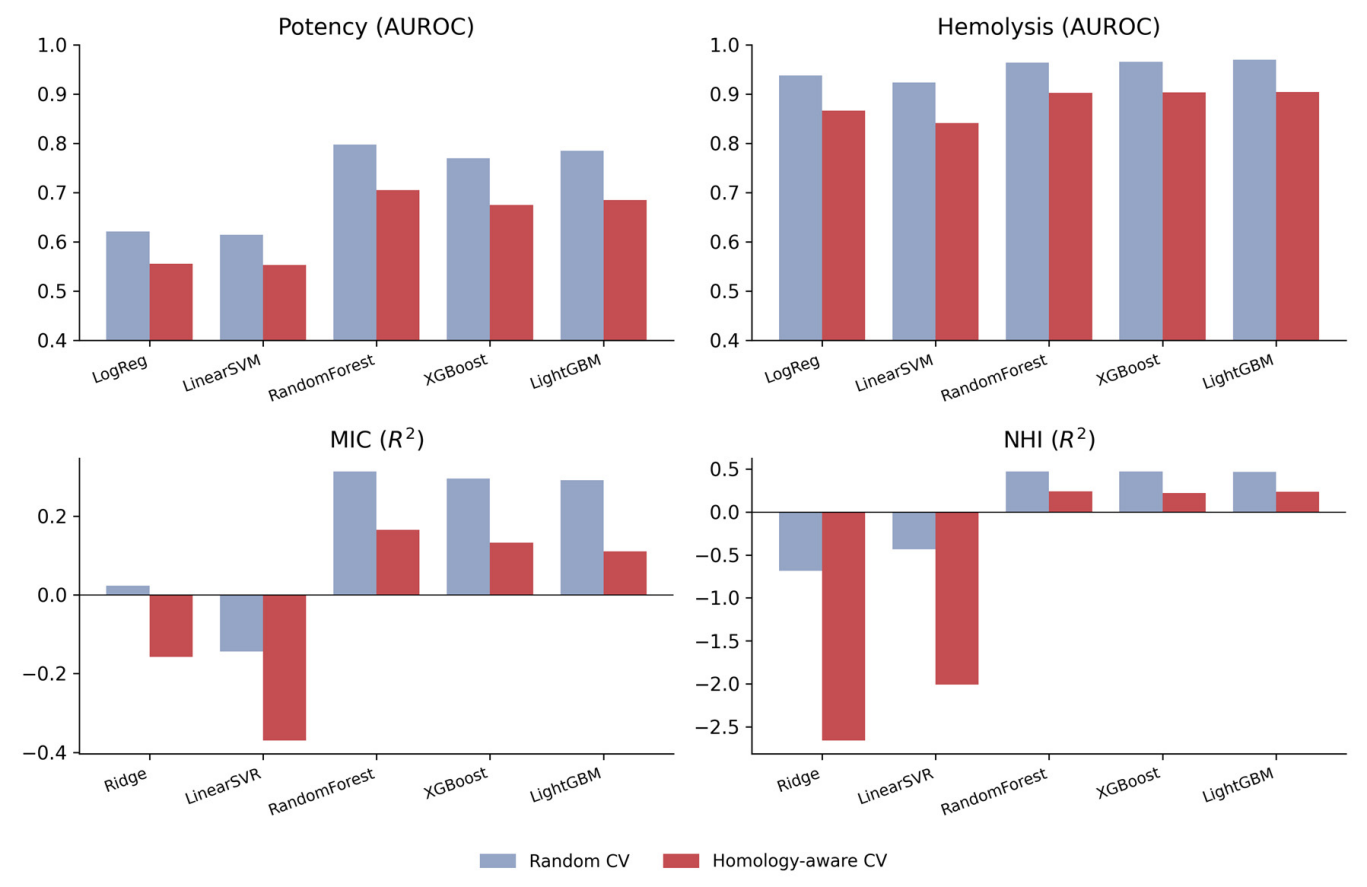
The same models re-evaluated under random versus homology-aware cross-validation. Bars show the primary metric per task (AUROC for classification, R^2^ for regression). Performance drops when scaffold-related sequences are kept out of the test folds, whereas hemolysis classification remains strong.

Figure 2 shows the five-fold cross-validation performance of all models under random splitting, and Table 1 reports every value under both random and homology-aware cross-validation, with the two schemes contrasted directly in Figure 3. Three points follow. First, homology-aware performance is uniformly lower, which confirms that random cross-validation is optimistic for this literature-derived data. The honest generalization estimate for potency classification is an AUROC of about 0.71 for Random Forest, compared with 0.797 under random splitting, and tree ensembles clearly exceed the linear baselines. Second, hemolysis classification remained strong under homology-aware validation, with a LightGBM AUROC of 0.90 (95% CI 0.88 to 0.93), a sensitivity of 0.79, and a specificity of 0.87. This demonstrates that its high accuracy is not a redundancy or leakage artifact. Third, MIC regression was modest (homology-aware R^2^ 0.17, Spearman ρ 0.39), and NHI regression was weaker still (R^2^ 0.24). We therefore treat MIC predictions as a rank-ordering signal only and do not interpret them as quantitative estimates. At the default 0.5 threshold, the potency classifier is highly sensitive but poorly specific, which is consistent with its use as a ranking tool rather than a hard classifier. Calibration curves are provided in the Supplementary Materials.

### 3.3. Structure–Activity Relationships

**Figure 4:**
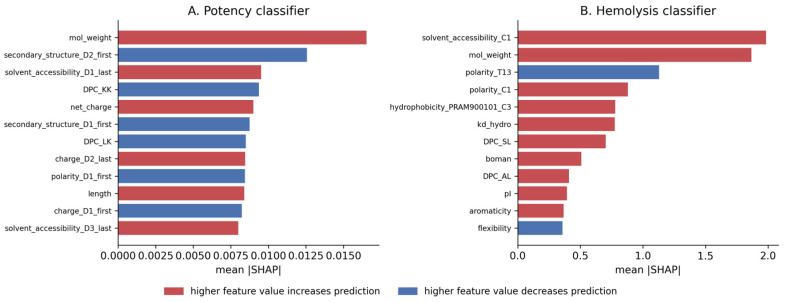
Top SHAP features for the production potency (**A**) and hemolysis (**B**) classifiers. A legend indicates the direction, positive or negative, of each feature’s contribution.

**Figure 5:**
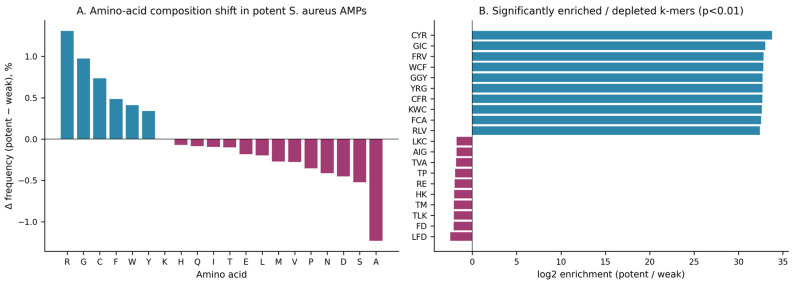
Amino-acid composition shift between potent and weak peptides (**A**) and Fisher’s exact k-mer enrichment (**B**); k-mers significant at *p* < 0.01 are shown, of which only the trimer CYR survives Bonferroni correction.

SHAP analysis of the potency classifier identified molecular weight, net positive charge, cationic dipeptides such as KK and LK, and secondary-structure and solvent-accessibility descriptors as leading contributors (Figure 4A). This is consistent with the classical cationic, amphipathic archetype of membrane-active AMPs [38]. Hemolysis was driven instead by bulk hydrophobicity and exposed non-polar surface (Figure 4B). Toxicity therefore tracks unpatterned hydrophobicity rather than being the simple inverse of potency, which supports the joint optimization of the two properties. Amino-acid composition shifts corroborated the SHAP profile, with enrichment of R, G, C, F, and W in potent sequences. Fisher’s exact testing identified the trimer CYR as the single k-mer surviving Bonferroni correction (adjusted *p* = 2.2 × 10^−3^; Figure 5), which points to cysteine-stabilized motifs [39,40]. These interpretable relationships are, in our view, the most robust output of the framework, because they do not depend on absolute predictive accuracy.

### 3.4. Multi-Objective Prioritization of Optimized Scaffolds

**Figure 6:**
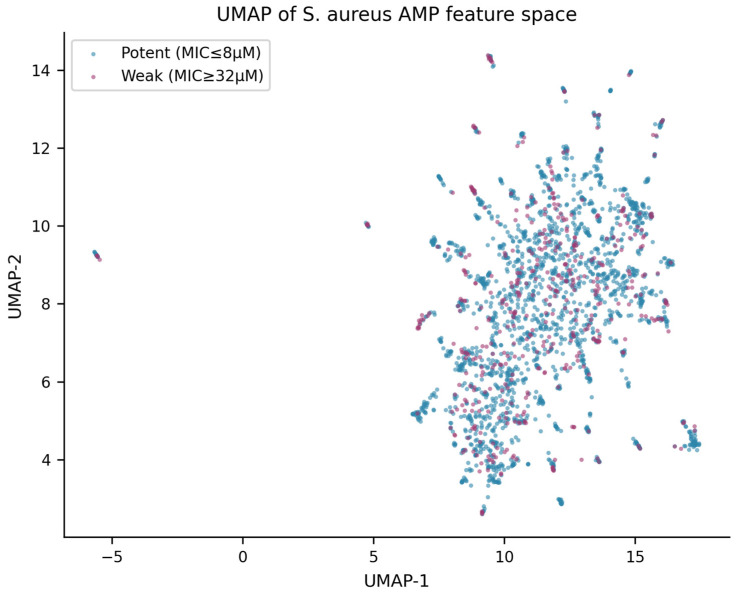
UMAP projection of the 538-dimensional feature space, colored by potency label. Potent and weak peptides overlap substantially, which is consistent with the moderate potency-classification performance. The projection is descriptive only and does not establish novelty or mechanism.

From the generated pool, the pipeline prioritized 20 candidates (Table 2). Nearest-neighbor analysis shows that these are optimized variants of known potent scaffolds rather than novel peptides. Their identity to the closest known peptide ranges from 80% to 100%, with a median of 95%, and the majority derive from a single high-potency 23-residue scaffold. We report these identities explicitly rather than claiming novelty from exact-match identity alone. The ranking is robust to the exact form of the scoring function. Perturbing the MIC scale and the component exponents preserves 16 to 20 of the top 20 and yields a Spearman ρ of at least 0.92 with the original ranking (Supplementary Table S5), which indicates that the specific heuristic constants do not drive the result. As expected, removing an objective entirely changes the ranking, which confirms that each objective contributes. Because candidates were filtered on predicted hemolysis, their near-zero predicted hemolysis reflects that selection rather than validated safety, and must be confirmed experimentally. A UMAP projection of the feature space (Figure 6) shows substantial overlap between potent and weak peptides, consistent with the moderate potency-classification performance. We present it as descriptive geography only and draw no novelty or mechanistic inference from it.

## 4. Discussion

Our results support a deliberately modest reading. Under honest, homology-aware evaluation, a compact interpretable model predicts anti-*S. aureus* potency with moderate accuracy (AUROC about 0.71) and orders peptides by relative potency, while a genuinely strong and homology-robust model (AUROC 0.90) flags likely hemolytic sequences. The contribution of this work is therefore not a new class of high-accuracy predictor, nor a set of novel peptides. It is threefold: an interpretable and quantitatively characterized structure–activity map for anti-staphylococcal activity versus hemolysis; a reliable hemolysis filter validated under homology-aware splitting; and a reproducible, sensitivity-checked multi-objective procedure that optimizes known potent scaffolds toward lower predicted toxicity, with candidate identities to known peptides reported transparently.

The interpretable structure–activity results are consistent with established AMP design principles [38,41]. Cationicity and amphipathicity drive *S. aureus* inhibition, whereas generalized hydrophobicity governs hemolysis, so that selecting against hemolysis need not sacrifice predicted potency. The recurrence of cysteine and the CYR motif is consistent with disulfide-stabilized families such as defensins [39,40].

Several limitations must be stated plainly. First, all findings are computational. No antimicrobial, antibiofilm, cytotoxicity, serum-stability, or synthesis experiments were performed, and the prioritized peptides are hypotheses for future testing. This work forms the computational first stage of a larger project. Wet-lab validation is ongoing and reported separately, and it is intentionally not claimed here. Second, the prioritized candidates are optimized variants of known scaffolds, with 80% to 100% identity to known peptides, so the framework performs lead optimization rather than de novo discovery. Third, the near-zero predicted hemolysis of the candidates is a consequence of the selection filter and of a hemolysis model trained on a separate, modest, and heterogeneous dataset that may not extrapolate to these cationic, tryptophan-rich sequences. It is not independent evidence of safety. Safety liabilities of new antimicrobials are often revealed only through post-marketing pharmacovigilance [42], which underscores the need for early and dedicated toxicity testing. Fourth, the model was trained only on anti-*S. aureus* activity and contains no cross-species information. It therefore models anti-staphylococcal potency and does not establish selectivity for *S. aureus* over other bacteria, which was not assessed. Fifth, MIC regression is weak and supports ranking only. Finally, avoiding protein-language-model embeddings favored interpretability at some cost in ceiling accuracy.

Beyond direct antibacterial use, membrane-active peptides such as these could in principle serve as recognition elements in peptide-based biosensors [43,44,45]. This remains a hypothesis that requires dedicated future work and is not a claim of the present study. Future work will pair the interpretable scoring developed here with experimental validation of a diversity-selected subset of candidates, will extend the models to strain-resolved subsets as data permit, and will explore formulation and delivery strategies, such as liposomal carriers for anti-MRSA agents [46], together with generative sampling for genuinely low-homology designs.

## 5. Conclusions

We developed an interpretable, homology-aware, multi-objective machine learning framework for prioritizing anti-*S. aureus* antimicrobial peptides. Reported honestly, it provides a moderate potency-ranking model, a strong and leakage-robust hemolysis filter, a transparent structure–activity map, and a reproducible procedure that optimizes known scaffolds toward lower predicted toxicity. The 20 prioritized peptides are experimentally testable hypotheses rather than validated leads, and we report their identity to known peptides transparently to avoid overstating novelty. We hope the emphasis on interpretability and on honest, homology-aware evaluation is useful to others who apply machine learning to antimicrobial-peptide prioritization.

## Figures and Tables

**Table 1:** Five-fold cross-validation under random versus homology-aware splitting.

Task	Model	Random	Homology-Aware	Secondary (Random/Homology)
**Potency classification**	Random Forest	0.797	**0.705**	MCC 0.346/0.214
**Hemolysis classification**	LightGBM	0.970	**0.904**	MCC 0.789/0.667
**MIC regression (log_10_)**	Random Forest	0.313	**0.165**	Spearman 0.503/0.389
**NHI regression**	Random Forest	0.468	**0.239**	Spearman 0.642/0.489

**Table 2:** Twenty prioritized candidates (optimized variants of known scaffolds). MIC is model-estimated and used for ranking only. P(hemo) is a selection criterion, not validated safety.

Sequence	Len	Net Charge	Amph. Moment	P(potent)	P(hemo)	MIC Est. (µM)	Score *D*	NN Identity	Nearest Known
**RIIDRLWRVRRPQKPKFVVVWVR**	23	+7.0	0.52	0.997	0.000	0.01	0.993	100%	dbAMP_23721
**RYIDLLWRQRRPQKPKFVIVWVR**	23	+6.0	0.37	0.997	0.001	0.07	0.978	100%	dbAMP_23721
**RKIDLLWRMRRPQKCKFFTVWVR**	23	+7.0	0.34	0.998	0.001	0.12	0.968	100%	dbAMP_23721
**RSIDRLWRCRRPQKPKFYIVWVR**	23	+7.0	0.46	0.982	0.000	0.08	0.962	100%	dbAMP_23721
**RIIDRLWMVRKNQKPKCVQYWVR**	23	+6.0	0.31	0.996	0.004	0.15	0.956	80%	dbAMP_33194
**RIIDRNWRVRRTQKPKFIIVWSR**	23	+7.0	0.42	0.981	0.001	0.12	0.952	100%	dbAMP_23721
**RIIDRLWRVRHPRKRKVVIHWVR**	23	+8.2	0.45	0.977	0.000	0.14	0.942	100%	dbAMP_23721
**RIIDRLQRVQRPQKPKFVHWWGR**	23	+6.1	0.61	0.978	0.000	0.20	0.930	80%	dbAMP_33190
**AWCFRVCYRGCCYRRCR**	17	+5.0	0.33	0.999	0.003	0.36	0.909	94%	dbAMP_17437
**HIIDRLPRVRRYQKPKIAIVWVR**	23	+6.1	0.43	0.977	0.000	0.30	0.907	80%	dbAMP_33190
**QIIDLRWRTRRNQKPKFVTVWYR**	23	+6.0	0.25	0.995	0.002	0.37	0.905	83%	dbAMP_23719
**RIIDRLWRKRRSQKPKRDIVWVR**	23	+8.0	0.49	0.932	0.000	0.14	0.899	100%	dbAMP_23721
**RWCFYARVRGVRYRRCW**	17	+6.0	0.18	0.996	0.000	0.42	0.896	100%	dbAMP_23719
**RHIDLLWWVRRPQKWKFCINHVR**	23	+5.2	0.37	0.997	0.001	0.52	0.875	83%	dbAMP_33341
**KLLKQWPIGKLLKKLLKKKLK**	21	+9.0	0.52	0.999	0.001	0.55	0.870	95%	dbAMP_05188
**RIIDILWRGRRPQHPKMVIHWVR**	23	+5.2	0.47	0.987	0.000	0.52	0.866	100%	dbAMP_23721
**RCIDRLWRVHRPQKPKMVTVAVR**	23	+6.1	0.60	0.984	0.000	0.52	0.865	83%	dbAMP_18104
**RIIDRLWRHGRPHKPKPVIVIVR**	23	+6.2	0.67	0.923	0.000	0.33	0.849	83%	dbAMP_18104
**VRKQPYLPRPRWPRRIYNR**	19	+7.0	0.35	0.997	0.004	0.66	0.842	95%	dbAMP_31695
**GIGAVLKWLPALFKRKRQQ**	19	+5.0	0.40	0.997	0.032	0.57	0.836	95%	dbAMP_18132

## Data Availability

The data, models, and scripts presented in this study are available within the article and its Supplementary Materials.

## Supplementary Materials

The following supporting information can be downloaded at: https://www.mdpi.com/article/10.3390/diagnostics16152430/s1, Table S1: complete filtered candidate list (top 2000 virtual-screening hits); Table S2: full SHAP feature-importance rankings for the potency and hemolysis classifiers; Table S3: complete feature-name list and full Fisher-exact k-mer enrichment table; Table S4: model diagnostics—confusion matrices, sensitivity, specificity, bootstrap 95% confidence intervals, the SMOTE-versus-class-weighting comparison, and calibration data; Table S5: sensitivity of the multi-objective score and the top-20 ranking to the scoring form; Table S6: per-candidate nearest-neighbor details (nearest known peptide and percent identity); Figure S1: physicochemical profile of the 20 prioritized candidates.

## Abbreviations

The following abbreviations are used in this manuscript:

AAC	Amino-Acid Composition
AMPs	Antimicrobial Peptides
AMR	Antimicrobial Resistance
AUPRC	Area Under the Precision–Recall Curve
AUROC	Area Under the Receiver Operating Characteristic Curve
CTD	Composition–Transition–Distribution
CV	Cross-Validation
DPC	Dipeptide Composition
GRAVY	Grand Average of Hydropathy
MCC	Matthews Correlation Coefficient
MIC	Minimum Inhibitory Concentration
MSSA	Methicillin-Susceptible *Staphylococcus aureus*
MRSA	Methicillin-Resistant *Staphylococcus aureus*
NHI	Normalized Hemolytic Index
SHAP	SHapley Additive exPlanations
SMOTE	Synthetic Minority Over-sampling Technique
UMAP	Uniform Manifold Approximation and Projection
